# Assessing the Effects of Surgical Irrigation Solutions on Human Neutrophil Interactions with Nascent *Staphylococcus aureus* Biofilms

**DOI:** 10.3390/microorganisms12101951

**Published:** 2024-09-27

**Authors:** Gauri Gaur, Maria Predtechenskaya, Jovanka M. Voyich, Garth James, Philip S. Stewart, Timothy R. Borgogna

**Affiliations:** 1 Department of Microbiology & Cell Biology, Montana State University, Bozeman, MT 59717, USA; gaurigaur@montana.edu (G.G.); mpredtec@montana.edu (M.P.); jovanka@montana.edu (J.M.V.); 2 Center for Biofilm Engineering, Montana State University, Bozeman, MT 59717, USA; gjames@montana.edu (G.J.); phil_s@montana.edu (P.S.S.)

**Keywords:** *S. aureus*, biofilm, neutrophil, irrigation, surgical site infection, implant, aggregates

## Abstract

*Staphylococcus aureus* (*S. aureus*) is the leading cause of surgical site infections (SSIs) and is capable of biofilm growth on implanted foreign devices. The use of surgical irrigation solutions has become a common strategy to combat bacterial contamination events that occur during surgery. Despite their antimicrobial activity, SSI rates remain consistent, suggesting that low-level contamination persists. In these cases, circulating neutrophils must traffic from the blood to contamination sites to aid in bacterial clearance. The influence of irrigation solutions on neutrophils’ ability to engage with bacteria has not been explored. The effects of three commonly used irrigation solutions: Xperience (sodium lauryl sulfate), Irrisept (chlorhexidine gluconate), and Betadine^®^ (povidone-iodine) on nascent *S. aureus* biofilms alone and in the presence of human neutrophils were assessed at manufactured and diluted concentrations. All three solutions, at a 10% dilution, inhibited bacterial growth as demonstrated by culture assays and confocal video microscopy of bacterial aggregate formation. The effects of 10% dilutions of each of these solutions on neutrophil membrane integrity (by flow cytometry and propidium iodide staining) and motility (by confocal video microscopy of neutrophil track length) were investigated with differing outcomes for each irrigation solution. At this concentration only Irrisept preserved neutrophil membrane integrity and motility. Together, this study examines an overlooked aspect of surgical irrigation solutions by investigating their impact on innate immunity and highlights the feasibility of formulations wherein solution effectiveness is complemented by neutrophil function to reduce risks of infection.

## 1. Introduction

The ability to combat infection, repair damage, and alleviate discomfort through surgical intervention is one of the triumphs of the modern medical era. While the benefits of most surgical interventions outweigh the associated risks, 1–5% of all surgeries are complicated by surgical site infections (SSIs) [1,2,3]. SSIs are defined as an infection occurring at the surgical site within 30 days of surgery [4,5]. Implantation of foreign materials can serve as scaffolding for bacterial biofilm growth and delay SSI detection for up to one year [5]. These infections often remain recalcitrant to interventions and require explantation for infection resolution. Collectively, patients affected by SSIs display an increased risk of mortality greater than 10-fold, expect an average extended hospitalization length of 9.7 days, and accrue an increased cost greater than USD 20,000 [4]. The substantial burden imposed by SSIs warrants evaluations and the development of strategies aimed at reducing the risk of SSI occurrence.

Bacterial contamination events causing SSIs most often occur at the time of surgery [6,7]. Unlike many hospital-acquired infections (HAIs) where etiologies were linked to exogenous sources such as hospital plumbing systems, poor instrument sterilization, or contaminated surfaces, during SSIs an individual’s own skin is often the primary reservoir for bacteria implicated in SSIs [6,8,9,10]. *Staphylococcus aureus* (*S. aureus*) is commonly described as a skin commensal but often serves as an opportunistic pathogen and was identified as the most frequent cause of SSIs [5,7,11]. *S. aureus* maintains a diverse array of immune evasion factors and has a high propensity for biofilm growth on abiotic surfaces [12,13]. In immune-competent hosts, infections with *S. aureus* are first confronted by neutrophils. Neutrophils are the most abundant leukocytes in circulation and are critical to early detection and clearance of staphylococcal infections [14,15]. Delayed neutrophil recruitment and subsequent *S. aureus* growth significantly impede bacterial clearance [16,17].

Efforts to prevent SSIs focus on the maintenance of a sterile environment during surgery. Given the threat of endogenous infection, eradication of possible contaminating organisms from the skin and incision are the predominant risk reduction strategies [18,19]. Antiseptic irrigation solutions are often used to flush the intraoperative space and aim to cleanse the wound from debris and contaminating microorganisms. This strategy is demonstrated to significantly reduce bacterial numbers; however, unless sterility is achieved, the risk of infection persists. In cases such as these, the prevention of SSIs may depend on the ability of circulating neutrophils to extravasate to the site of contamination and destroy remaining bacteria through potent intra- and extracellular killing mechanisms. Thus, irrigation solutions that inhibit bacterial growth while maintaining an environment permissive to neutrophil function may enhance the clearance of contaminating microorganisms. 

In this study, we assessed three irrigation solutions for bactericidal capacity in the presence of human neutrophils. Betadine (povidone-iodine) was first introduced as an antiseptic in 1955 and remains the most widely used surgical site irrigation solution [20,21]. Iodine solutions function as potent oxidizers and cause severe damage to bacterial cell membranes, proteins, and nucleic acids [22]. While this mechanism of action kills a broad spectrum of microorganisms, its effects are not restricted to surgical contaminants and host cell toxicity is often observed [21,22,23]. Irrisept is an antimicrobial wound lavage containing 0.05% chlorhexidine gluconate [24]. Chlorhexidine gluconate was first developed in the 1940s and was recognized for displaying antibiofilm activity in the 1970s [25]. Cationic chlorhexidine molecules destabilize bacterial cell walls through charge–charge interactions leading to a disruption in osmotic equilibrium and cell death [22,25]. These solutions are demonstrated to reduce bacterial numbers of organisms commonly associated with SSIs and are reported to cause minimal host cell damage [22,24,26,27]. XPerience (XP) is a novel irrigation solution formulated with citric acid, sodium citrate, and sodium lauryl sulfate [24]. These components function in combination by disrupting biofilm matrices and lysing exposed bacterial cells. In contrast to other irrigation solutions, XP is designed as a “no-rinse” solution, suggesting XP confers minimal off-targeted toxicity to host cells; however, no studies have examined the effects of XP on neutrophils.

While the concern of irrigation solution toxicity on host cells and leukocytes was raised, there is a paucity of data examining these effects on primary leukocytes. To date, no studies have directly investigated the effects of these solutions on neutrophils [28]. Using confocal microscopy, we directly visualized interactions between *S. aureus* nascent biofilms and human neutrophils in the presence of irrigation solutions. Collectively, these data demonstrate the plausibility of irrigation solution formulations that retain antimicrobial activity while remaining conducive to neutrophil function. Moreover, our findings suggest irrigation solution effectiveness may be complemented by neutrophil-mediated bacterial killing, leading to fewer contamination events and an overall reduction in SSI occurrences. 

## 2. Materials and Methods

### 2.1. Bacterial Strains

*Staphylococcus aureus* (*S. aureus*) strain AH2547 was used in these studies. This strain constitutively expresses green fluorescence protein (GFP) through the addition of pCM29 to methicillin-susceptible strain *S. aureus* HG001 [17]. Nascent *S. aureus* biofilms were grown and attached as described in Ghimire et al. [16]. Briefly, overnight cultures of *S. aureus* were grown in tryptic soy broth (TSB) supplemented with 10 µg/mL of chloramphenicol. Aliquots were centrifuged, washed, and serial diluted in Dulbecco’s Phosphate-Buffered Saline (DPBS) to adjust the optical density (OD_600_) to 0.1. To attach bacteria to 96-well (Greiner Bio-one, Monroe, NC, USA) flat bottom plates or four-chambered glass bottom petri dishes (Cellvis, Mountain View, CA, USA), 10 µL of the diluted bacteria was applied directly to the surface followed by incubation at 37 °C for 30 min. At the end of the 30 min incubation, unattached bacteria were removed by gently rinsing in DPBS. Hank’s Balanced Salt Solution with Ca^2+^ and Mg^2+^ (HBSS), XPerience (XP) (Next Science LLC, Jacksonville, FL, USA), Irrisept (Irrimax Corporation, Lawrenceville, GA, USA), or Betadine (Avrio Health L.P., Stamford, CT, USA) was added to each well at the appropriate percentage and supplemented with 10% of freshly isolated autologous normal human serum (NHS). 

### 2.2. Neutrophil Preparations

Heparinized venous blood from healthy donors was collected in accordance with an Institutional Review Board for Human Subjects approved protocol at Montana State University. Human neutrophils were isolated under endotoxin-free conditions as previously described [14,29]. Neutrophil purity (<2% PBMC contamination) and viability (<5% propidium iodide positive) were assessed using BD FACS Calibur flow cytometer.

### 2.3. Irrigation Solution Titration Experiments

*S. aureus* AH2547 nascent biofilms were attached to 96-well plate bottoms as described above. Solutions were serially diluted 1:2 from 100% to 0.1% in HBSS containing a final in-well concentration of 10% NHS. Control wells contained HBSS + 10% NHS only. All wells contained a final volume of 100 µL. Dark-walled 96-well plates (Greiner Bio-one, Monroe, NC, USA) were used to capture relative fluorescent intensities. A Cytation 5 Imaging reader (BioTek, Winooski, VT, USA) was used to quantify GFP fluorescence at 37 °C for four hours at 5-min intervals by excitation at 485 nm and emission at 528 nm. Bacterial growth was determined by GFP detection over time. Minimum inhibitory concentrations (MICs) were identified with a custom protocol based on detected changes in the overall GFP fluorescence. MIC was taken as the lowest solution percent resulting in <2.5-fold increase in GFP detection following four-hour incubations. Fold increase was calculated by dividing the GFP fluorescence intensity at four hours by the GFP fluorescence intensity at time zero.

### 2.4. Microscopy

A Leica DMi8 inverted confocal laser scanning microscope was utilized for all imaging. An Okolab (Ambridge, PA, USA) Uno Stage Top Incubator with a stand-alone humidity controller was utilized to maintain 5% CO_2_, 20% O_2_, 90% humidity, and 37 °C for sample incubation and during imaging. For all microscopy studies, experiments were performed in four-chambered glass-bottom petri dishes. For experiments analyzing solution effects on aggregate number and size following four-hour growth, images of each well were collected using a Leica 10×/0.4 NA dry objective lens to obtain merged 4 × 5 tile scans with a z-stack height of 30 µm with 3 µm z-intervals. GFP-tagged bacteria were excited with a 488 nm laser.

For microscopy experiments using human neutrophils, the neutrophils were kept on ice after isolation until stained with LysoBrite™ Red (AAT Bioquest, Pleasanton, CA, USA, Cat no. 22645) according to the manufacturer’s instructions. In neutrophil challenge experiments, the diluted bacteria were grown in the four-chambered dish with 10% of the appropriate solution and 10% NHS for four hours at 37 °C, 5% CO_2_, and 90% humidity. Post the four-hour incubation, the LysoBrite™ Red stained neutrophil cells were immediately added to each chamber surface followed by the addition of 5 µg/mL propidium iodide (PI). Cells stained with LysoBrite Red were excited with a 590 nm laser and the damaged cells stained with PI were excited with a 510 nm laser along with additional TauGating to differentiate the excitation time for the different neutrophil stainings. Image stacks (20 µm) with 1 μm z-slices were recorded sequentially at 5 minute intervals over a four-hour time course using a Leica 20×/0.7 NA dry objective lens. One field of view was imaged per chamber in each experiment. 

To enumerate the surviving bacteria, bacteria were detached from the chamber-well surface by repeated pipetting of the well contents. The solutions were removed from the wells, placed in 1.5 mL tubes, and sonicated for 5 min at 60 Hz in a bath sonicator. Following sonication, tubes were vortexed for 30 s before being serially diluted. Diluted bacteria were plated on tryptic soy agar (TSA) and incubated overnight at 37 °C with 5% CO_2_. Resulting colonies from the overnight incubations were manually enumerated. 

### 2.5. Image Analysis

At the end of the four-hour incubation, merged 4 × 5 tile scan images were analyzed as maximum projection z-stacks. MetaMorph v 7.8.13 (Molecular Devices) image analysis software was used as described in Pettygrove et al. to quantify aggregate object numbers [30]. The Integrated Morphometry Analysis tool was used to measure the object diameter and area.

Time-lapse movies were prepared with Imaris version 10.01 (Oxford Instruments, Abingdon, UK). For the analysis of individual neutrophil–bacteria interactions, the Imaris Spots feature was used to identify neutrophils and PI staining events. The tracks of the neutrophils were mapped in each condition and track length was calculated using the average total length of the neutrophil displacements within the track.

### 2.6. Flow Cytometry

Neutrophil viability experiments were completed in 96-well flat bottom plates that were pretreated with autologous NHS for 30 min and rinsed prior to the addition of neutrophils. Neutrophils were resuspended at 1 × 10^7^ cells/mL in HBSS and 100 µL was aliquoted into wells. Cells were allowed to settle for 5 min. Irrigation solutions were diluted and added to appropriate wells. All wells were supplemented with 10% NHS. Plates were incubated at 37 °C for one hour. Following incubation, the contents of each well were transferred into flow cytometry tubes. Cells were washed and stained with Zombie Violet (Pac Blue) viability dye (BioLegend) at a 1:250 dilution for 20 min at room temperature in the dark. After staining, cells were washed, resuspended in a final volume of 300 µL of FACS buffer, and immediately analyzed. All neutrophil flow cytometry experiments were performed on a BD LSR Fortessa and resulting FCS files were analyzed using FlowJo 10.8.1. 

## 3. Results

### 3.1. Irrigation Solutions Inhibit the Growth of S. aureus at Low Concentrations

**Figure 1:**
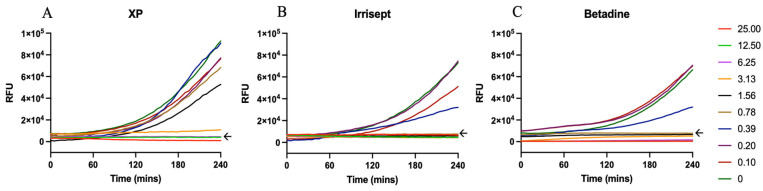
Irrigation solutions below 10% manufacturer concentrations inhibit nascent *S. aureus* biofilm growth. *S. aureus* aggregates were attached to wells in 96-well plates. Aggregates were incubated for four hours in irrigation solutions serially diluted 1:2 in HBSS supplement with 10% NHS. GFP mean fluorescence intensity was detected over time (**A**) XPerience, (**B**) Irrisept, or (**C**) Betadine. Data displayed are representative of three biological replicates.

Antiseptic surgical irrigation solutions are formulated to combat microbial contamination and can be applied during preoperative, operative, and postoperative procedures [3,31,32]. Though effective at microbial killing, manufactured concentrations are often toxic to host cells and are frequently diluted prior to use [33,34,35]. To that end, we first sought to identify the lowest concentration that retained growth inhibitory effects (Figure 1). Inhibitory growth concentrations of XP, Irrisept, and Betadine towards *S. aureus* nascent biofilms were measured using an adaptation of the biofilm growth model described in Ghimire et al. [16]. Briefly, small *S. aureus* aggregates were pre-attached to multiwell plates followed by incubation in each solution and corresponding dilution for four hours. Consistent with reports and directed uses, the use of all solutions at manufactured concentrations resulted in no detectible growth of *S. aureus* (Figure 1). As expected, serial dilutions of each irrigation solution resulted in a concentration-dependent loss of growth inhibition. The minimum inhibitory concentration (MIC), expressed as a percentage of the full-strength solution, was 6.25% for XP and 0.78% for Irrisept and Betadine (Figure 1A–C) (Supplemental Table S1). Notably, all solutions impacted bacterial growth at <10% of the manufactured concentrations.

### 3.2. Visualization and Quantification of Irrigation Solution Effects on Nascent S. aureus Biofilms

**Figure 2:**
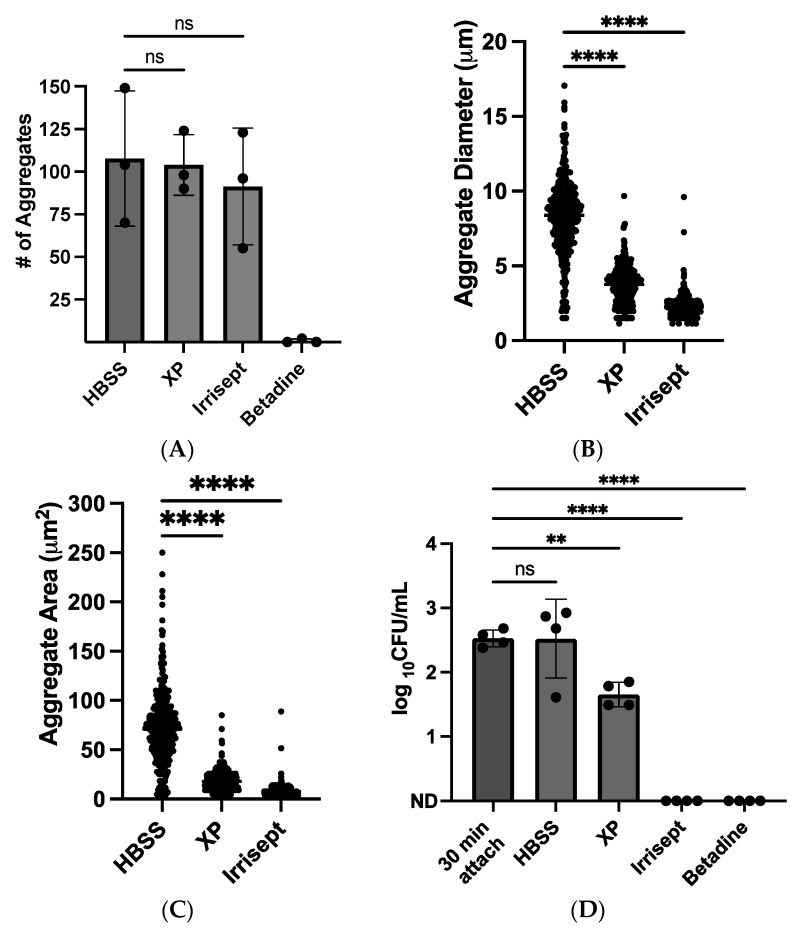
Direct visualization and quantification of 10% irrigation solution on *S. aureus* growth. Confocal images were taken 30 min post-attachment and following four-hour growth in the specified condition. Maximum projection z-stack quantification of (**A**) total aggregates detected (**B**) aggregate diameter and (**C**) aggregate area. Data are from three biological replicates. (**D**) Colony forming units (CFUs) of *S. aureus* after four-hour growth in solutions. Colonies were manually counted following overnight growth on TSA (ND = not detected). Data displayed are from four separate experiments. ns: not significant ** *p* < 0.005, **** *p* < 0.0001 as analyzed by one-way ANOVA followed by Dunnett’s multiple comparisons test. Error bars indicate mean ± SD.

After identifying that the 10% solutions inhibited or suppressed *S. aureus* growth, we sought to directly visualize the effects of these irrigation solutions on nascent biofilms at this concentration. Using confocal microscopy, time-lapse images of *S. aureus* growth in corresponding solutions were collected for four hours as described in Pettygrove et al., 2021 [17]. In the HBSS control solution, the number of bacterial objects or aggregates remained stable over the observation period while the size of aggregates increased due to bacterial growth (Figure 2B,C). No significant changes in aggregate numbers were observed when comparing the HBSS control solution to Irrisept and XP solutions at four hours (Figure 2A). In contrast, incubation in the 10% Betadine solution eliminated nearly all visible GFP signals and abolished aggregate growth. Only two aggregates were detected in one of three biological replicates. The total number of detected objects did not significantly differ among the XP, Irrisept, and HBSS control solutions; however, the average aggregate diameter and area were significantly smaller in XP (3.7 µm, 18.4 µm^2^) and Irrisept (2.2 µm, 8.3 µm^2^) solutions as compared to the HBSS (8.2 µm, 72.1 µm^2^) control (Figure 2B,C and Figure S1). In these experiments, treatment with Irrisept consistently resulted in smaller aggregate formation compared to both the control and XP solution. Taken together, these data suggest that treatment of *S. aureus* nascent biofilms with 10% solutions of XP and Irrisept resulted in bacteriostatic effects, whereas treatment with 10% Betadine was bactericidal. To ensure that the reduced bacterial detection in diluted irrigation solutions was due to growth inhibition and not interference of the solutions with GFP detection, viable colony plate counts were obtained following incubation with irrigation solutions or control for four hours (Figure 2D). Colony forming units (CFUs) collected from these experiments confirmed that the 10% concentrations of all solutions significantly impact bacterial growth as compared to the control (Figure 2D and Figure S2).

### 3.3. Neutrophil Membrane Integrity at Low Irrigation Solution Concentrations

The timely migration of circulating neutrophils to infection sites is essential for the clearance of *S. aureus* [15,17]. Recent in vitro studies demonstrated that neutrophil killing of *S. aureus* is significantly impaired if bacterial aggregates have a chance to enlarge prior to neutrophil arrival [16,17,36]. The relatively consistent rate of SSI occurrence, despite the application of irrigation solutions, implies that adequate disinfection of the intraoperative space is not always achieved. In cases where small numbers of contaminating organisms persist, the prevention of an SSI is influenced by host immune responses and the ability of neutrophils to find and kill remaining contaminant bacteria. Taken together, we hypothesized that irrigation solutions that are capable of arresting growth or disrupting biofilm formation while maintaining an environment conducive to neutrophil function may be key to wound disinfection and preventing SSIs.

**Figure 3:**
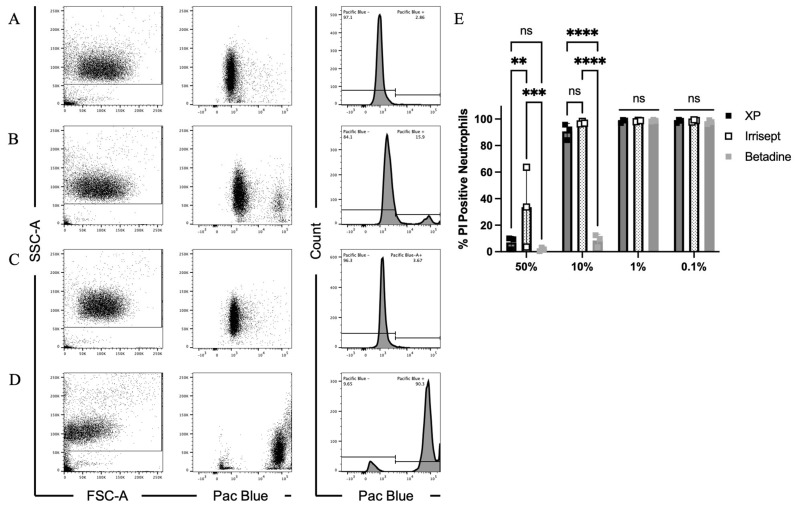
Effects of irrigation solutions at low doses on neutrophil plasma membrane damage. Flow cytometric analysis of neutrophil membrane integrity. Neutrophils were incubated in 10% irrigation solutions. Representative plots showing forward (FSC-A) and side scatter (SSC-A) or membrane permeability as measured by increase in Pac Blue staining (dot and histogram plots) of (**A**) HBSS only, (**B**) XP, (**C**) Irrisept, or (**D**) Betadine. (**E**) Quantification of neutrophil plasma membrane damage following incubation with irrigation solutions. Data displayed are from three biological replicates. ** *p* < 0.005, *** *p* < 0.0005, **** *p* < 0.0001 as determined by two-way ANOVA followed by Tukey’s multiple comparisons test. Error bars indicate mean ± SD.

To begin to explore the possible complementary contributions of neutrophils towards bacterial clearance, we first assessed the effects of the irrigation solutions on neutrophil viability by measuring plasma membrane damage. Neutrophils were incubated for one hour in varied doses of each irrigation solution. Following incubation, flow cytometry was used to quantify the intracellular accumulation of Zombie Violet (Pac Blue), a membrane-impermeable amine-reactive dye (Figure 3 and Figure S3). Treatment of human neutrophils with all solutions at 0.1% and 1% concentrations resulted in average viability across replicates >98% (Figure 3E and Figure S3). At the 10% concentration, XP and Irrisept solutions had minimal effects on neutrophil membrane permeability and retained an average viability >90% (Figure 3A–C,E). Despite no effects on membrane integrity, a slight cellular condensation was observed for the 10% XP-treated neutrophil population as indicated by a small shift forward scatter (FSC-A) [37]. In accordance with previous studies, treatment with 10% Betadine severely disrupted neutrophil membrane integrity (increase in Pac Blue fluorescence) resulting in plasma membrane damage causing drastic shifts in both cell size and granularity and reducing viability to an average of 8.5% (Figure 3D,E) [21,38].

Given the minimal toxicity observed for XP and Irresept at 10% concentrations, we were unsure if concentrations more closely resembling the commercially manufactured concentrations would negatively impact neutrophil viability. To directly address the toxicity of commercially manufactured concentrations, irrigation solutions and neutrophils were combined at equal volumes yielding an in-well solution concentration of 50%. These samples were incubated for one hour followed by analysis using flow cytometry. Incubation of neutrophils in XP, Irrisept, and Betadine in 50% of the commercially manufactured concentrations caused significant plasma membrane damage. Intact plasma membranes were observed in only 7%, 33.6%, and 1.7% of neutrophils treated with the respective solutions (Figure 3E and Figure S3). Collectively, these data demonstrate that 10% Betadine solution is damaging to human neutrophils; however, 10% XP and Irrisept solutions do not significantly compromise neutrophil membrane integrity. Whereas these data strongly imply incubation in 10% XP or Irrisept solutions is not cytotoxic to neutrophils, they do not address impacts on function such as neutrophil motility or bacterial killing.

### 3.4. Neutrophil Motility and Engagement of Bacteria in Irrigation Solutions

**Figure 4:**
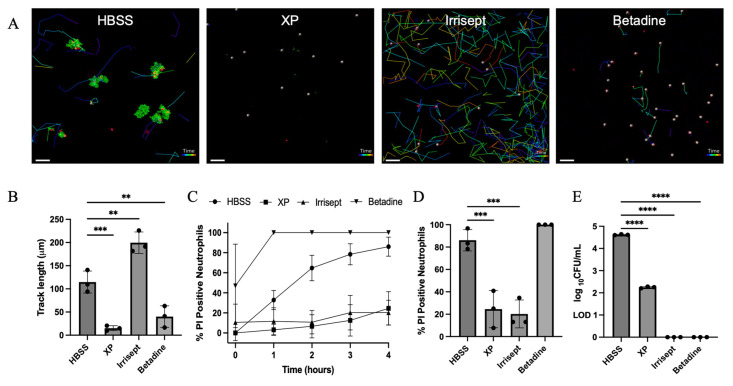
Neutrophil motility, bacterial engagement, and clearance in 10% irrigation solutions. (**A**) Representative images of the neutrophil track length following four-hour incubation of *S. aureus* in HBSS, XP, Irrisept, or Betadine, respectively. Neutrophils stained with LysoBrite Red (blue) and propidium iodide (PI, red) following four-hour interactions with *S. aureus* aggregates (green) grown with 10% concentration of the displayed irrigation solution with 20 μm z-stacks. (**B**) Quantification of neutrophil track lengths post four-hour incubation in respective solutions. (**C**) Accumulation of PI-positive neutrophils during interactions with nascent biofilms in irrigation solutions. (**D**) PI-positive neutrophils after four-hour interactions with nascent biofilms in irrigation solutions. (**E**) Quantification of remaining *S. aureus* CFUs following four-hour growth in solutions in the presence of neutrophils (ND = not detected). LOD = 10 CFU/mL. Scale bar = 50 μm. Images displayed are representative of one field of view and of three biological replicates. ** *p* < 0.005, *** *p* < 0.001, **** *p* < 0.0001 as analyzed by one-way ANOVA with Dunnett’s multiple comparisons test. Error bars indicate mean ± SD.

Using time-lapse confocal microscopy, we assessed neutrophil motility and discovery of bacteria in 10% irrigation solutions (Figure 4). To best replicate the application and sequence of irrigation solution used, nascent biofilms were first incubated in the presence of each 10% irrigation or control solution for four hours (Figure 2 and Figure S1). At four hours post aggregate growth, neutrophils were added to each chamber. Propidium iodide (PI) was aliquoted into the chambers and images were collected at five-minute intervals for an additional four hours (Videos S1–S4). After four hours neutrophil motility was quantified in all solutions (Figure 4A,B). For these experiments, motility was expressed as the total track length, which is defined as the average distance traveled by all neutrophils within one condition.

While mobilization of neutrophils to sites of infection is a requisite of function, the presence of neutrophils is not a correlate of bacterial clearance or wound healing. For these reasons, we directly visualized neutrophil interaction with *S. aureus* aggregates. Consistent with previous studies, Neutrophils are highly motile in HBSS but were unable to effectively kill bacterial aggregates given the size of aggregates due to the head-start growths [17] (Figure 4B). Large bacterial aggregates were resistant to neutrophil offenses and caused significant membrane damage as evidenced by the accumulation of PI-positive neutrophils at the aggregate interface (Figure 4A,C,D, Video S1) [17]. Despite no effects on membrane permeability, treatment with a 10% XP solution abolished neutrophil motility and resulted in no interaction with remaining *S. aureus* aggregates (Figure 4A–D, Video S2). Treatment with 10% Irrisept had no negative effects on neutrophil motility (Figure 4B, Video S3). Indeed, neutrophils readily patrolled and engaged remaining bacterial aggregates with minimal accumulation of PI in neutrophils contacting aggregates (Figure 4A,C,D). Treatment with a 10% Betadine solution caused significant damage to neutrophil membranes (Figure 3D,E). Not surprisingly, these cells demonstrated no motility and were immediately positive for PI (Figure 4A–D, Video S4). Bacteria CFUs were enumerated from each chamber following four-hour incubations with neutrophils (Figure 4E) and confirmed previous observations that a significant reduction in bacteria was observed in all irrigation solutions compared to HBSS control. Taken together, these data demonstrate that a 10% Irrisept solution can promote bacterial clearance by disrupting bacterial growth while maintaining aspects of neutrophil function. Moreover, these findings highlight an overlooked strategy to combat SSIs wherein enhanced bacterial clearance is achieved through formulations of irrigation solutions that promote synergism with host immune defenses.

## 4. Discussion

In this study, we investigated whether the antibacterial effects of three commonly used irrigation solutions could be complemented by neutrophil-mediated bacterial killing. Given the high prevalence and severity of SSIs resulting from *S. aureus* contamination on implanted surfaces, our study focused on irrigation solution effectiveness during the early interactions between *S. aureus* and neutrophils. Using an established model of neutrophil interactions with nascent *S. aureus* biofilms on abiotic surfaces, we directly compared the effects of commonly used irrigation solutions on bacterial growth and neutrophil clearance [16,30].

Irrigation solutions target contaminating microbial organisms and are demonstrated safe for topical use. Despite this, off-target host cell toxicity is reported at commercially manufactured concentrations [21,22,23,26]. Given these reports and our emphasis on neutrophil health in irrigation solutions, we sought to identify a maximum concentration common among the irrigation solutions that inhibited bacterial growth while preserving neutrophil viability. Furthermore, despite the effects of solutions on planktonic cultures being well-defined, the potency of these solutions against nascent *S. aureus* biofilms remained understudied. To center on a productive solution concentration for these studies we first diluted each solution and measured *S. aureus* growth by detection of GFP. All solutions were highly effective at inhibiting *S. aureus* growth well below the commercially manufactured concentration. Following this initial concentration screening, 10% solutions were chosen for further analysis due to similar potencies at this concentration across all the solutions. It should be noted that while irrigation solutions are at times diluted prior to use, the low concentration of 10% used in these studies may not be indicative of in vivo efficacies.

The effects of each 10% solution on nascent biofilms were visualized using confocal microscopy. In these experiments, treatment with Betadine largely reduced bacterial aggregates below the threshold of detection. Among three independent biological replicates, only two distinct *S. aureus* aggregates were detected. Given the potency of Betadine and the inability to recover bacterial CFUs from any of these replicates, we are confident that the reduction in the number of aggregates is due to the bactericidal activity of Betadine under these conditions. Both XP and Irrisept are utilized for their antibiofilm properties [24,39]. Following four-hour incubations, no significant changes in the number of aggregates were detected between these solutions and the control; however, aggregate size was significantly reduced. Reductions in CFUs recovered from wells containing XP were observed. CFUs were unable to be recovered from wells treated with Irrisept. Given that aggregates were detected in these samples, it is more likely that treatment with Irrisept reduced bacterial numbers below the limit of detection rather than resulted in sterilization of the well.

The reduced bacterial aggregate size caused by XP and Irrisept solutions highlights a fundamental aspect of complemented bacterial clearance by neutrophils. Neutrophils are highly adapted to engage and kill *S. aureus* at low numbers and small aggregate sizes [16,36]. The effectiveness of neutrophil-mediated *S. aureus* clearance is largely determined by the multiplicity of infection (MOI) [12,40]. Increases in *S. aureus* cell numbers or aggregate size can overwhelm neutrophil offensives leading to continued bacterial growth. *S. aureus* aggregates ≥50 µm^2^ not only resist neutrophil killing but demonstrate an enhanced ability to lyse nearby neutrophils [16,36]. Taken together, the ability of these solutions to restrict bacterial growth likely enhances neutrophil efficiency in these environments.

In order for neutrophils to contribute to bacterial clearance in the presence of irrigation solutions, host cell toxicity must be minimized. We used flow cytometry to assess neutrophil membrane permeability in each solution. At the 10% concentration, both XP and Irrisept preserved neutrophil membrane integrity, whereas treatment with Betadine caused significant membrane damage. Given previous studies examining the effects of Betadine on multiple cell types, off-target toxicity from povidone-iodine-induced oxidative stress from these solutions was not surprising [21,22,41,42]. The preservation of membrane integrity observed in the XP and Irrisept solutions suggested that the 10% concentration may not affect neutrophil motility or function.

Despite no significant increases in membrane permeability, we noted a slight reduction in the FSC-A of the neutrophil population treated with 10% XP indicating a reduction in cell size. Commercial manufactured solutions of XP maintain a pH of ~ 4.0 and it was unaltered by reducing the concentration to 10%. Consistent with our observations, studies examining the impact of low pH environments on neutrophils have reported a condensation in cell size and a reduction in motility [43,44]. Time-lapse imaging analysis of neutrophils in the presence of the XP solution demonstrated that while some neutrophils remain viable, nearly all neutrophils were immobile (Figure 4A, Video S2). To confirm the reductions in neutrophil motility observed in the 10% XP solutions were due to the acidic pH, solutions were titrated with NaOH to pH 7.0 representing a more optimal pH for neutrophils. Neutralization of the XP solutions restored neutrophil motility but abolished bacteriostatic effects (Video S5).

In direct contrast to the reduced neutrophil motility observed following XP treatment, treatment with Irrisept increased neutrophil motility metrics above the HBSS control. Consistent with previous reports, head-start bacterial growth in the HBSS for four hours leads to *S. aureus* aggregates >50 µm^2^ [16,17,36]. At this size, leukocidins likely accumulate at the aggregate surface at concentrations that interfere with neutrophil efficacy and lead to toxin-mediated neutrophil cell death. Treatment with Irrisept restricted *S. aureus* growth. At four hours, all observed aggregates remained at <50 µm^2^ and were unable to cause neutrophil damage. As a result of these effects, neutrophils readily surveyed the abiotic surface and engaged the remaining aggregates. The reduced bacterial growth and increased neutrophil motility conferred by treatment with 10% Irrisept demonstrate the feasibility and possible benefits of host-permissive irrigation solutions.

In the current study, the effects of Irrisept on neutrophil-mediated bacterial killing were not directly explored. It is possible that neutrophil killing mechanisms such as the generation of the phagolysosome and production of reactive oxygen species were negatively impacted. However, the ability of neutrophils to successfully chemotax and interact with remaining aggregates without significant increases in PI uptake suggests the neutrophil function is unchanged. Future studies will directly test the functionality and efficiency of neutrophil bacterial killing mechanisms in chlorhexidine gluconate solutions. In summary, these findings highlight an overlooked strategy to combat SSIs wherein enhanced bacterial clearance is achieved through formulations of irrigation solutions that promote synergism with host immune defenses.

## 5. Conclusions

The premise behind this study was that an ideal surgical irrigation solution would combine antimicrobial properties with innate immune system compatibility. A solution that is bacteriostatic or bactericidal while also preserving the normal function of frontline phagocytic cells has logical appeal for an infection control application. We explored the potential of this concept by measuring the effects of three commercial irrigation solutions on bacterial growth as well as on human neutrophil viability and motility. Even in this preliminary investigation with just three products, we discovered a range of qualitative outcomes. Betadine exhibited bacteriostatic and bactericidal activity but strongly permeabilized neutrophil cell membranes and strongly suppressed neutrophil motility. The potent antimicrobial activity of this solution may be offset in vivo by its interference with neutrophil function. XP exhibited modest inhibition of bacterial growth without reducing neutrophil viability but eliminated neutrophil motility. This solution, therefore, may also suppress innate immune cell performance. Irrisept combined effective inhibition of bacterial growth while maintaining neutrophil viability and motility.

These diverse outcomes suggest that it may be valuable and important to assess compatibility with innate immune system function when developing antimicrobial or antibiofilm products. Two technologies with similar antimicrobial activity, one of which compromises innate immune cell function and another that is innate immune compatible, could plausibly have differing efficacy in the clinic. Of course, this conjecture will ultimately need to be validated with animal models and human clinical data.

## Data Availability

The original contributions presented in the study are included in the article/Supplementary Materials, further inquiries can be directed to the corresponding author.

## Supplementary Materials

The following supporting information can be downloaded at https://www.mdpi.com/article/10.3390/microorganisms12101951/s1, Figure S1: Nascent *S. aureus* biofilm growth in 10% irrigation solutions; Figure S2: CFUs recovered following attachment and growth in irrigation solutions; Figure S3: Irrigation solution effects on neutrophil membrane permeability; Table S1: Mean fold-change in GFP intensity at four hours; Video S1: Representative time-lapse videos showing neutrophil interactions with *S. aureus* aggregates in HBSS. Video S2: Representative time-lapse videos showing neutrophil interactions with *S. aureus* aggregates in 10% XP. Video S3: Representative time-lapse videos showing neutrophil interactions with *S. aureus* aggregates in 10% Irrisept. Video S4: Representative time-lapse videos showing neutrophil interactions with *S. aureus* aggregates in 10% Betadine. Video S5: Representative time-lapse showing neutrophil interactions with *S. aureus* aggregates in 10% XP adjusted to a pH of 7.0 using NaOH.
