# Supplementary material for: Assessing the Effects of Surgical Irrigation Solutions on Human Neutrophil Interactions with Nascent Staphylococcus aureus Biofilms
Source: Microorganisms. 2024 Sep 27;12(10):1951. doi: 10.3390/microorganisms12101951 (PMC11509154; doi:10.3390/microorganisms12101951)
Supplement: Supplementary file 1 [file microorganisms-12-01951-s001.zip › Gaur_et_al_supplemental_submitted.pdf]

Table S1. Mean fold-change in GFP intensity at four hours.
at four hours.

Solution	XP	Irrisept	Betadine
**25.00%**	0.44	1.18	1.08
**12.50%**	1.55	1.06	1.09
**6.25%**	2.15*	0.97	4.61
**3.13%**	3.30	0.97	6.97
**1.56%**	6.96	0.95	1.38
**0.78%**	9.27	1.06*	1.21*
**0.39%**	11.04	3.04	5.76
**0.20%**	8.62	4.76	7.06
**0.10%**	8.75	6.70	7.16
**0.00%**	8.77	7.69	8.14

*Indicates MIC

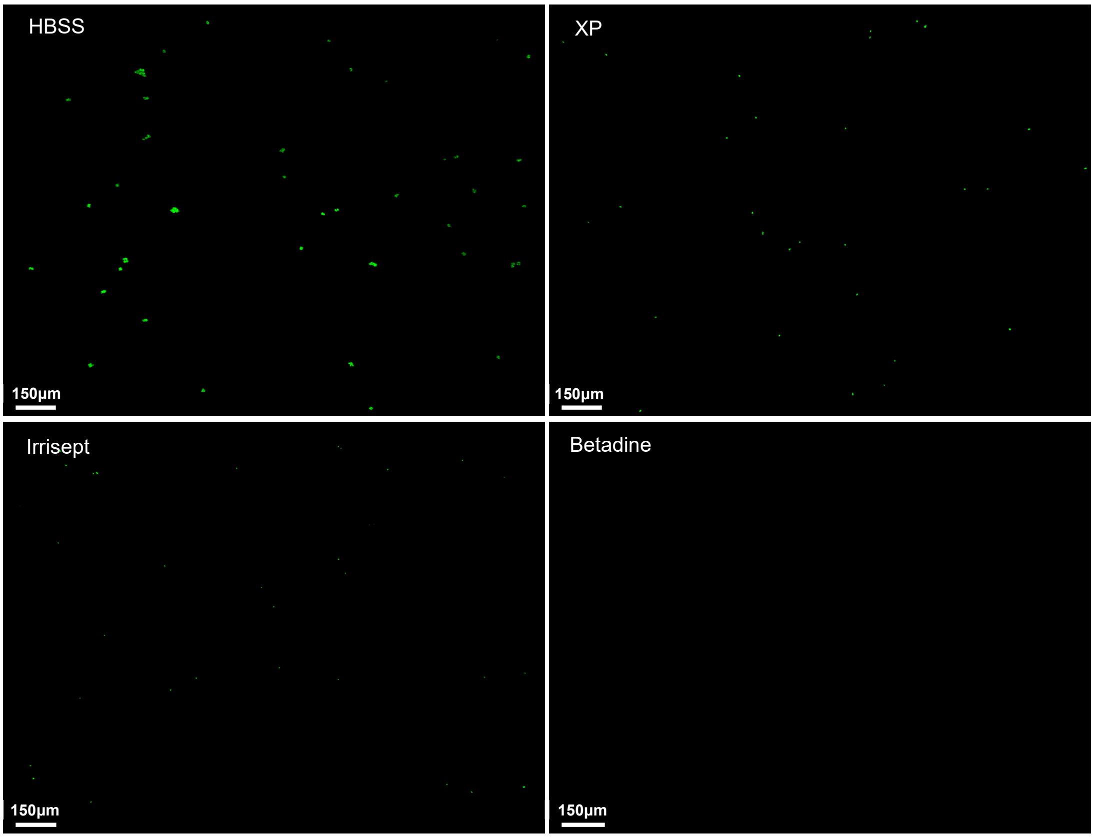

Supplemental Figure 1: Nascent *S. aureus* biofilm growth in 10% irrigation solutions. Maximum intensity projection of 30µm z-stacks following a four-hour incubation with 10% concentration of the indicated solution. Scale bar = 150µm. Images shown are of one field of view and are representative of three biological replicates.

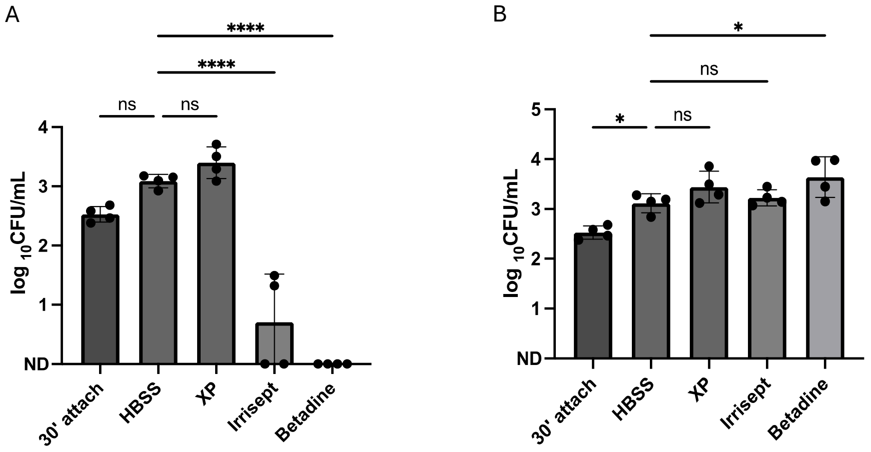

Supplemental Figure 2. CFUs recovered following attachment and growth in irrigation solutions. CFUs recovered 30 minutes post-attachment and following four-hour growth in the specified conditions A) irrigation solutions diluted to 1% manufactured concentration, B) irrigation solutions diluted to 0.1% manufactured concentration. Colonies were manually counted following overnight growth on TSA (ND = not detected). Data are from four separate biological replicates *$p < 0.05$, ****$p < 0.0001$ by one-way ANOVA followed by Dunnett's multiple comparisons test. Error bars indicate mean ± SD.

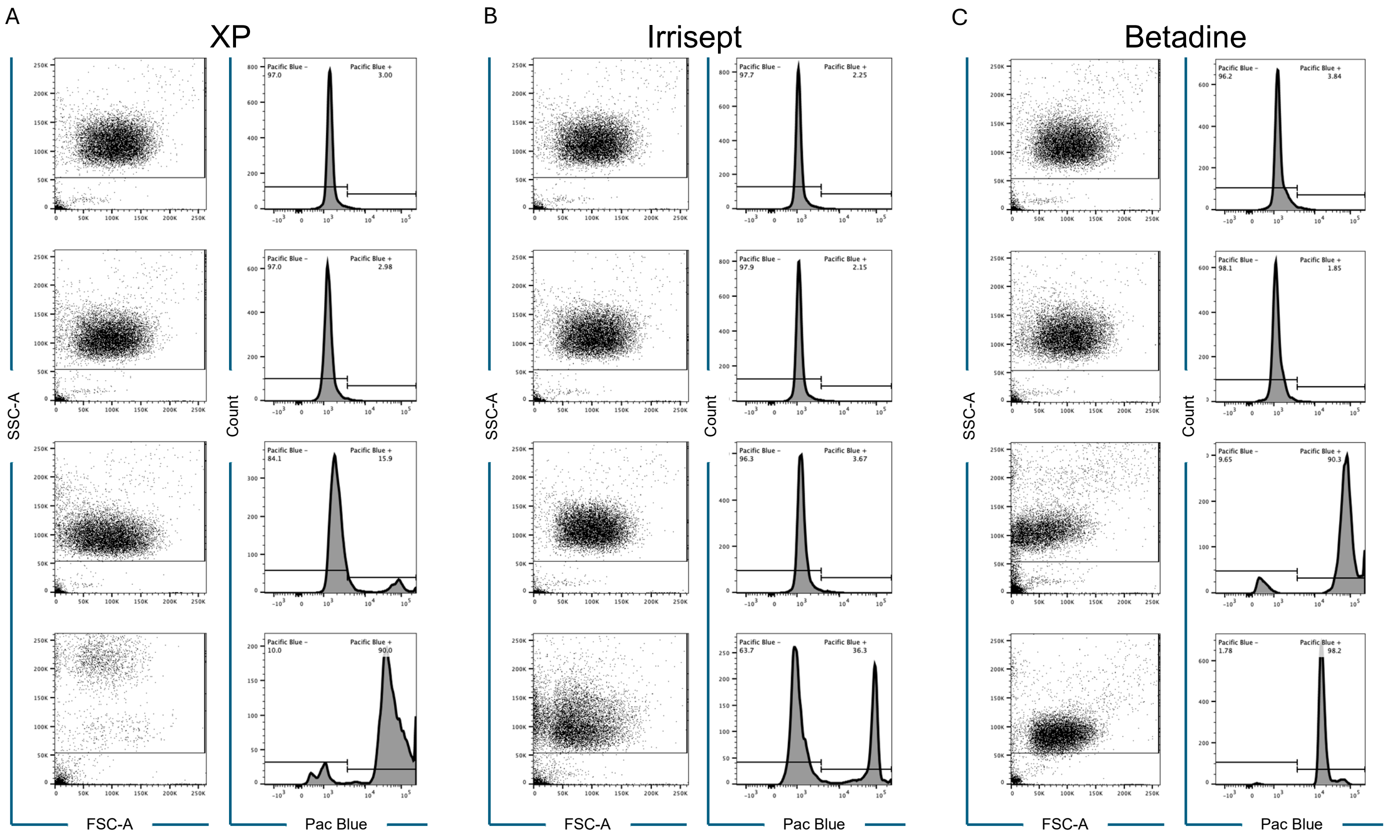

Supplemental Figure 3. Irrigation solution effects on neutrophil membrane permeability. Flow cytometric analysis of neutrophil membrane integrity. Neutrophils were incubated in 50%, 10%, 1%, and 0.1% irrigation solutions for one hour.
