# Supplementary material for: Assessing the Effects of Surgical Irrigation Solutions on Human Neutrophil Interactions with Nascent Staphylococcus aureus Biofilms
Source: Microorganisms. 2024 Sep 27;12(10):1951. doi: 10.3390/microorganisms12101951 (PMC11509154; doi:10.3390/microorganisms12101951)
Supplement: Supplementary file 1 [file microorganisms-12-01951-s001.zip › Gaur_et_al_supplemental_video_legends.pdf]

Video S1: Representative time-lapse videos showing neutrophil interactions with *S. aureus* aggregates in HBSS. *S. aureus* aggregates were grown in HBSS solution for four hours. Neutrophils were added to the solution and images were recorded at five-minute intervals for an additional four hours. Scale bar = 50 μm.

Video S2: Representative time-lapse videos showing neutrophil interactions with *S. aureus* aggregates in 10% XP. *S. aureus* aggregates were grown in XP solution for four hours. Neutrophils were added to the solution and images were recorded at five-minute intervals for an additional four hours. Scale bar = 50 μm.

Video S3: Representative time-lapse videos showing neutrophil interactions with *S. aureus* aggregates in 10% Irrisept. *S. aureus* aggregates were grown in Irrisept solution for four hours. Neutrophils were added to the solution and images were recorded at five-minute intervals for an additional four hours. Scale bar = 50 μm.

Video S4: Representative time-lapse videos showing neutrophil interactions with *S. aureus* aggregates in 10% Betadine. *S. aureus* aggregates were grown in Betadine solution for four hours. Neutrophils were added to the solution and images were recorded at five-minute intervals for an additional four hours. Scale bar = 50 μm.

Video S5: Representative time-lapse showing neutrophil interactions with *S. aureus* aggregates in 10% XP adjusted to a pH of 7.0 using NaOH. *S. aureus* aggregates were grown in the pH-adjusted 10% XP solution for four hours. Neutrophils were added to the solution and images were recorded at five-minute intervals for an additional four hours. Scale bar = 50 μm
